# A simplified description of X-ray free-electron lasers

**DOI:** 10.1107/S090904951004896X

**Published:** 2011-01-08

**Authors:** G. Margaritondo, Primoz Rebernik Ribic

**Affiliations:** a Faculté des Sciences de Base, Ecole Polytechnique Fédérale de Lausanne (EPFL), CH-1015 Lausanne, Switzerland

**Keywords:** free-electron laser, SASE, X-ray laser

## Abstract

It is shown that an elementary semi-quantitative approach explains essential features of the X-ray free-electron laser mechanism, in particular those of the gain and saturation lengths. Using mathematical methods and derivations simpler than complete theories, this treatment reveals the basic physics that dominates the mechanism and makes it difficult to realise free-electron lasers for short wavelengths. This approach can be specifically useful for teachers at different levels and for colleagues interested in presenting X-ray free-electron lasers to non-specialized audiences.

## Abstract 2

An elementary derivation of fundamental properties of X-ray free-electron lasers is presented, including gain and saturation. Because of its simplicity, this approach is particularly suitable for teaching at different levels and for presentations to non-specialized audiences.

## 1. Motivation

X-ray free-electron lasers (X-FELs) are finally a reality: the recent success of the Stanford Coherent Light Source (LCLS) (Emma *et al.*, 2010 ▶) is attracting considerable attention worldwide, not limited to the directly involved community nor to physics. This makes it desirable to have a theoretical treatment accessible to non-specialists and students. Past experience with synchrotron sources (Margaritondo, 1988 ▶, 1995 ▶, 2002 ▶) indicates that an effort in this direction may enhance the use of the new machines, extend it to new research communities and facilitate teaching tasks at different levels.

We present here what is, we believe, the simplest description so far of the X-FEL mechanism. Without complicated formalism, we can explain the role of relevant factors. The underlying physical phenomena become easily understandable, in particular what makes it difficult to build lasers for X-rays.

Note that because of the relativistic velocity of the electrons in the X-FEL, such phenomena are not intuitive. For example, we shall see that the optical amplification depends on the electrons forming microbunches with a space period close to the emitted wavelength. Why, then, is the effect much more difficult to achieve for short X-ray wavelengths than for visible light? On the contrary, one could imagine that microbunching is easier to obtain if the distance between microbunches is shorter! We shall see how relativity explains this apparent paradox.

## 2. Qualitative description

**Figure 1:**
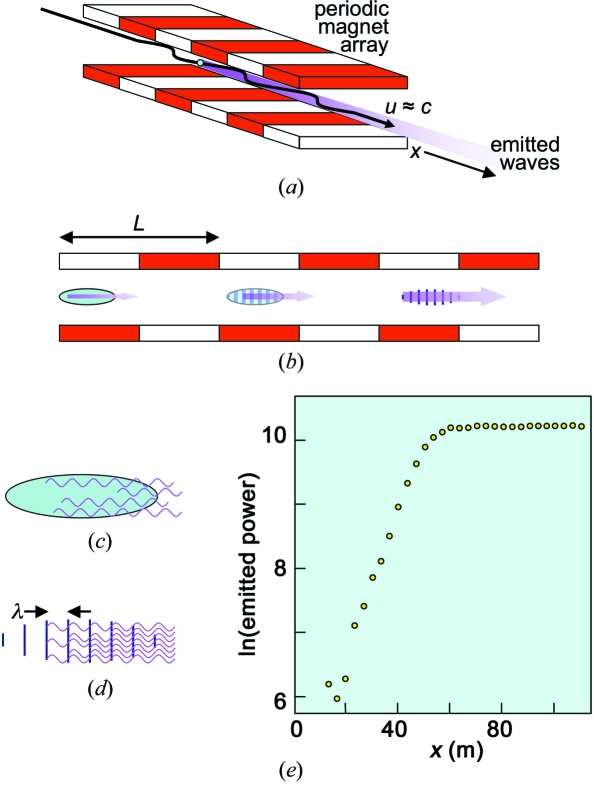
Mechanism of a free-electron laser for X-rays. (*a*) The optical amplification is produced by relativistic electrons in an accelerator and is activated by a periodic array of magnets (undulator). (*b*) The first waves emitted by the electrons trigger the formation of microbunches. (*c*) and (*d*) Contrary to non-microbunched electrons (*c*), the emission of electrons in microbunches (*d*) separated from each other by one wavelength is correlated. (*e*) This causes an exponential intensity increase with the distance that continues until saturation is reached as discussed in the text [experimental data from Emma *et al.* (2010 ▶)].

Fig. 1 ▶ schematically explains how an X-FEL works (Madey, 1971 ▶; Dattoli & Renieri, 1984 ▶; Dattoli *et al.*, 1995 ▶; Patterson *et al.*, 2010 ▶; Bonifacio *et al.*, 1984 ▶, 1994 ▶; Bonifacio & Casagrande, 1985 ▶; Pellegrini, 2000 ▶; Murphy & Pellegrini, 1985 ▶; Kim, 1986 ▶; Huang & Kim, 2007 ▶; Kim & Xie, 1993 ▶; Brau, 1990 ▶; Kondratenko & Saldin, 1980 ▶; Milton *et al.*, 2001 ▶; Schmueser *et al.*, 2008 ▶; Feldhaus *et al.*, 2005 ▶; Altarelli, 2010 ▶; Shintake, 2007 ▶; Shintake *et al.*, 2003 ▶; Roberson & Sprangle, 1989 ▶; Saldin *et al.*, 2000 ▶). The optical amplification takes place within electron bunches traveling inside a linear accelerator (LINAC) at a (longitudinal) speed *u* ≃ *c*, the speed of light. The emission and amplification of electromagnetic waves are activated by a periodic magnet array (‘undulator’) with period *L*. The undulator magnetic field can be written as *B* = *B*_0_sin(2π*x*/*L*) = *B*_0_sin(2π*ut*/*L*). Subject to this field, the electrons slightly undulate with a periodic transverse velocity component *v*_T_. These oscillations and the corresponding acceleration cause the electron charges to emit electromagnetic waves.

In a normal undulator source the electrons emit electromagnetic waves without correlation with each other (Fig. 1*c* ▶) and the total intensity is the sum of the intensities produced by individual electrons, proportional to *N*/Σ, the number of electrons in the bunch divided by the bunch cross section. If *i* is the electron beam current corresponding to the electron bunch in the accelerator, then *N*/Σ is proportional to *i*/Σ.

In an X-FEL [Figs. 1(*b*) and 1(*d*) ▶] the electrons emit in a correlated way (Emma *et al.*, 2010 ▶; Dattoli & Renieri, 1984 ▶; Dattoli *et al.*, 1995 ▶; Huang & Kim, 2007 ▶). Assume that a given electron, after entering the undulator, emits a wave. The (transverse) *B*-field of this wave and the transverse velocity of the electrons create a longitudinal Lorentz force that pushes the electrons to form microbunches with a periodicity *equal to the emitted wavelength*. The electrons within a microbunch oscillate all together under the effect of the undulator, and their wave emission is correlated (Fig. 1*d* ▶). The *E*-field (or the *B*-field) of the waves emitted by individual electrons are added together, rather than their intensity.

This has two consequences: (i) since the wave intensity is proportional to the square of the *E*-field, the total emitted intensity is proportional to *N* ^2^ rather than to *N*; (ii) the total wave intensity is progressively amplified along the undulator (Fig. 1*e* ▶) according, as we shall see, to an exponential law (Emma *et al.*, 2010 ▶; Huang & Kim, 2007 ▶).

The amplification does not continue indefinitely: saturation occurs after a distance *L*_S_ (Fig. 1*e* ▶). One criterion in designing an X-FEL is to reach saturation before the end of the undulator (Emma *et al.*, 2010 ▶). In most lasers the path available for amplification is expanded by an external optical cavity. This is not possible for X-rays since normal-incidence mirrors are extremely ineffective at the corresponding wavelengths. Hence, a ‘one-pass’ strategy is required, with strong amplification and a very long undulator.

Note that the starting wave subsequently amplified could be an external X-ray beam injected along with the electron beam (a ‘seed’) rather than the spontaneous initial emission of the electrons (Huang & Kim, 2007 ▶). In that case the laser works as an amplifier rather than as a self-contained source. When spontaneous initial emission is used, the mechanism is called SASE (self-amplified spontaneous emission) (Bonifacio *et al.*, 1984 ▶).

## 3. What causes an exponential intensity increase?

This property can be discussed even before analyzing the details of the X-FEL mechanism. The amplification is due to the energy transfer from the electrons to the previously emitted wave. This requires a *negative* work of the force caused by the wave (transverse) *E*-field (note that the *B*-field cannot do any work).

The time rate of energy transfer for one electron is proportional to the product *E*_W_ *v*_T_, the wave *E*-field magnitude times the electron transverse velocity. In turn, *E*_W_ is proportional to the square root of the wave intensity, thus the energy transfer rate from each electron is proportional to *I* ^1/2^ *v*_T_. Therefore, the uncorrelated combination of the effects of individual electrons would not correspond to an exponential increase of the intensity with the distance but to a quadratic law.

Microbunching changes this by forcing the electrons to emit in a correlated way. What causes microbunching? As we already mentioned, microbunching is caused by the inter­action between the electrons oscillating in the transverse direction and the transverse *B*-field of the previously emitted waves. Indeed, the transverse velocity and the *B*-field produce a longitudinal Lorentz force that, as we shall discuss in detail later, pushes the electrons to form microbunches.

The microbunching Lorentz force is proportional to the transverse electron velocity and to the wave *B*-field strength *B*_W_. Since *B*_W_ is proportional to the square root of the wave intensity, the microbunching force is proportional to *I* ^1/2^.

How does microbunching influence the subsequent wave emission? Let us assume that it enhances the correlated emission by a factor proportional to the microbunching force, an assumption that we will justify later. Multiplied by the energy transfer rate for each electron, this factor gives d*I*/d*t* = *AI* with *A* = constant, corresponding indeed to an exponential intensity increase along the undulator.

Assuming *A* = *u*/*L*_G_, we obtain the commonly used form (Bonifacio *et al.*, 1984 ▶; Huang & Kim, 2007 ▶) for the exponential intensity law,
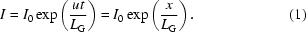
The parameter *L*_G_, called ‘gain length’, characterizes the amplification and the corresponding requirements to obtain lasing.

The functional form of (1) is verified experimentally (Emma *et al.*, 2010 ▶). Therefore, we will use it for the rest of our discussion as an empirical fact.

## 4. Emission by individual electrons

We now summarize some basic features of the emission of an electron traveling in an undulator (Margaritondo, 2002 ▶) that are valid, in particular, for an X-FEL, and explain fundamental properties such as the emitted wavelength. Since the electron speed is (almost) the speed of light *c*, the treatment is based on special relativity.

**Figure 2:**
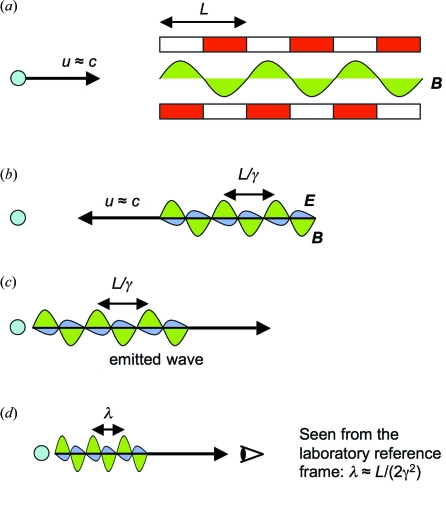
Why are the emitted wavelengths in the X-ray range? Relativity provides the answer. (*a*) The relativistic electron approaches the periodic *B*-field of the undulator. (*b*) In the electron reference frame the undulator period *L* is Lorentz-contracted to *L*/γ and the *B*-field is accompanied by a transverse *E*-field perpendicular to it: the two fields resemble an electromagnetic wave. (*c*) This wave stimulates the electron to oscillate and emit waves of equal wavelength. (*d*) The (relativistic) Doppler effect further reduces the wavelength in the laboratory frame, bringing it to the X-ray range.

In the electron reference frame, the undulator transverse *B*-field (Fig. 2*a* ▶), after a Lorentz transformation, becomes the combination of a transverse *B*-field plus a transverse *E*-field (Fig. 2*b* ▶), traveling together at a speed *u* ≃ *c*. These are also the characteristics of an electromagnetic wave. The wavelength of this wave is given, in the electron reference frame, by the undulator period corrected for the relativistic Lorentz contraction. In the longitudinal direction the contracted length is *L*/γ, where γ is the relativistic γ-factor, defined by the equation 1/γ ^2^ = (1 − *u* ^2^/*c* ^2^) and proportional to the electron energy γ*m*_0_ *c* ^2^ (*m*_0_ = electron rest mass).

The electron, therefore, ‘sees’ the undulator as an electromagnetic wave (Fig. 2*b* ▶). This wave causes the electron to oscillate and to emit waves of equal wavelength. Thus, the emitted wavelength in the electron reference frame is *L*/γ.

However, seen in the laboratory reference frame (Fig. 2*c* ▶) the wavelength emitted by the moving electron must be further corrected for the longitudinal Doppler effect. The additional correction factor is ∼2γ, so that the wavelength becomes
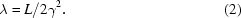
According to (2), to obtain X-rays the macroscopic undulator period *L* must be downscaled by many orders of magnitude using a large γ. Thus, an X-FEL requires a high-energy accelerator.

Equation (2) is not entirely correct since it does not take into account the impact on γ of the undulator *B*-field that induces the electron transverse velocity. The Lorentz force causing *v*_T_ cannot do any work: it cannot modify the electron kinetic energy and the overall velocity magnitude. The presence of *v*_T_ thus causes a decrease in the longitudinal velocity, to values < *u*. The effective 1/γ^2^ factor in (2) becomes larger than (1 − *u* ^2^/*c* ^2^) and depends on *B*.

It is easy to demonstrate that the corresponding corrected form of (2) is
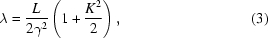
where the so-called ‘undulator parameter’ *K* is proportional to the maximum undulator *B*-field strength *B*_0_ and to *L*. In fact, owing to electron kinetic energy conservation, the longitudinal speed squared decreases from *u* ^2^ to (*u* ^2^ − *v*_T_^2^). Thus, in (2), 1/γ^2^ changes to 1 − (*u* ^2^ − *v*_T_^2^)/*c* ^2^ = (1/γ^2^)(1 + *v*_T_^2^γ^2^/*c* ^2^). This is consistent with (3) since, as we shall see later, *v*_T_ is proportional to *B*_0_ *L*/γ. Note that (3) implies that the emitted wavelength of an X-FEL can be controlled by changing the undulator *B*-field strength.

In a real undulator, and in an X-FEL, the emission occurs not at one wavelength but in a wavelength band of width Δλ around the central value defined by (3) [or, in first approximation, by (2)]. This bandwidth can be estimated by taking into account that each electron going through the undulator emits a wave train consisting of a number of wavelengths equal to the number of undulator periods, *N*_u_. The time duration Δ*t* of this pulse is the pulse length divided by the speed of light, *N*_u_λ/*c*.

According to the Fourier transforms, a pulse of duration Δ*t* has a frequency bandwidth Δν = 1/Δ*t*; thus, Δν = *c*/(*N*_u_λ). Wavelength and frequency are related as ν = *c*/λ, which by differentiation gives Δν = *c*Δλ/λ^2^, thus Δλ = Δνλ^2^/*c* = λ/*N*_u_ and
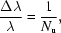
a relative wavelength bandwidth decreasing as the number of undulator periods increases.

## 5. Factors influencing the gain length and the amplification

We will now discuss in detail the mechanism illustrated in Fig. 1 ▶. Note that a rigorous theoretical treatment is intrinsically complicated even in the simplest one-dimensional case (Bonifacio *et al.*, 1984 ▶). It leads to a third-order differential equation whose solution is the combination of three terms. One of them dominates during the exponential amplification and justifies it. The exponential amplification is preceded by a preliminary phase with a slower intensity build-up, and is followed by the saturation phase.

We do not try to tackle all these fine theoretical aspects, but explain with simple arguments their qualitative and quantitative consequences, starting from amplification. Remember that the rate of energy transfer from an individual electron to the pre-existing wave is proportional to *I* ^1/2^ *v*_T_. Thus, to find the amplification we must evaluate *v*_T_. However, the total correlated emission intensity from *all* electrons also depends on microbunching; thus, to find the amplification we must also evaluate the degree of microbunching.

We start with *v*_T_ that is caused (Fig. 1 ▶) by the undulator *B*-field. For transverse-motion dynamics, the relevant equation is Newton’s law with the relativistic mass,
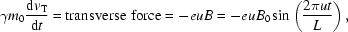
which gives
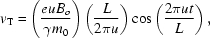
which is proportional to (*B*_0_ *L*/γ). Thus, the energy transfer rate by a single electron is proportional to *I* ^1/2^(*B*_0_ *L*/γ). We will leave out for now the cosine factor, for reasons that will be clarified later.

As to microbunching, the longitudinal microbunching force is proportional to *v*_T_ and to the wave *B*-field (pictured in Fig. 2 ▶). In turn, the wave *B*-field is proportional to the square root of the wave intensity, and therefore [see (1)] to 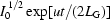. The microbunching force can then be written as
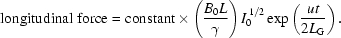
This force induces a small longitudinal electron displacement Δ*x* superimposed on the average motion with speed *u*. For longitudinal dynamics the relevant relativistic equation is derived from the general law that the time derivative of the longitudinal momentum γ*m*_0_(dΔ*x*/d*t*) equals the longitudinal force. The result (neglecting the small transverse oscillations) is
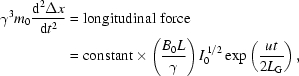
where the factor γ^3^ *m*_0_ is the so-called relativistic ‘longitudinal mass’. After integration, the above equation gives a longitudinal displacement towards microbunching,
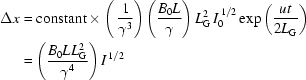
(note that we assumed a negligibly small initial wave intensity for Δ*x* = 0 m, where the amplification and motion towards microbunching start).

Maximum microbunching means that the electrons are concentrated in narrow slabs separated from each other by a distance equivalent to the wavelength λ. The degree of microbunching, corresponding to the fraction of electrons that emit in a correlated way, can be assumed in a first approximation to be proportional to (Δ*x*/λ). The corresponding number of electrons is proportional to *N*(Δ*x*/λ). Their contribution to the wave intensity is proportional to (*i*/Σ)(Δ*x*/λ), in turn proportional [see (2)] to (*i*/Σ){[(*B*_0_ *LL*_G_^2^/γ^4^)*I* ^1/2^]/(*L*/γ^2^)} = (*i*/Σ)(*B*_0_ *L*_G_^2^/γ^2^)*I* ^1/2^.

These arguments justify our previous assumption that microbunching effects correspond to a factor proportional to the longitudinal microbunching force and therefore to *I* ^1/2^. In addition, they reveal other important elements in this factor. Multiplying the factor by the energy transfer rate for one electron, we see that the total transfer rate is proportional to
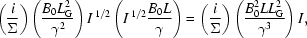
and we can write
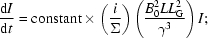
this is, indeed, an equation of the form d*I*/d*t* = *AI*, whose solution is (1) as long as *u*/*L*_G_ (≃ c/*L*_G_) is proportional to (*i*/Σ)(*B*_0_^2^ *LL*_G_^2^/γ^3^), or
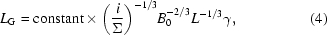

            *i.e.* a result consistent with those (Bonifacio *et al.*, 1984 ▶; Huang & Kim, 2007 ▶) of rigorous and complete theories and with their conceptual physics foundations.

This result can be expressed in terms of the ‘FEL parameter’ or ‘Pierce parameter’ ρ, corresponding to
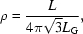
introduced by Bonifacio *et al.* (1984 ▶), and linked to the most important FEL properties. Equation (4) thus implies
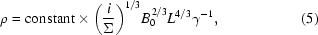
in agreement with its rigorous theoretical definition.

Equations (4) and (5) put in evidence essential factors that keep the gain length short, as required for an X-FEL. First, the undulator parameters *B*_0_ and *L* must be maximized, keeping in mind, however, that *L* also determines the wavelength. The electron beam current must be high and its transverse cross section small. However, the γ-factor cannot be freely decreased if we want to obtain X-ray wavelengths [see equations (2) and (3)].

## 6. Microbunching: electrons and waves traveling together

**Figure 3:**
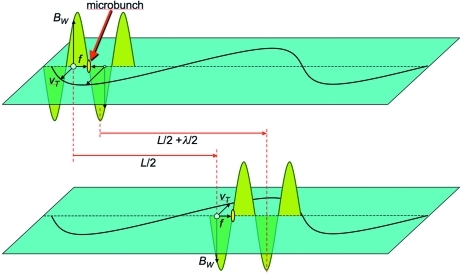
The speed difference (*c* − *u*) between waves and electrons makes microbunching possible. Top: in this situation the longitudinal Lorentz forces caused by the wave *B*-field *B*_W_ and to the electron transverse velocity *v*_T_ push the electrons towards microbunching. Bottom: after the electron travels over one-half undulator period, its transverse velocity is reversed. The wave travels ahead of the electron by one-half wavelength: its *B*-field is also reversed, the Lorentz force keeps its direction and microbunching continues.

So far we have not considered the sine and cosine factors in the transverse velocity and in the wave. This can be justified *a posteriori*, based on the fact that the electron microbunching occurs only because of some subtle effects that merit additional analysis (see Fig. 3 ▶). Assume that at a certain time (Fig. 3 ▶, top) the *B*-field of the already existing wave and the electron transverse velocity *v*_T_ create a Lorentz force *f* pushing the electron towards a wave node. This can indeed lead to microbunching.

Imagine, however, that electron and wave travel together with exactly the same speed. After one-half of the undulator period the electron transverse velocity would be reversed whereas the wave *B*-field would keep the same direction. The Lorentz force would be reversed and the microbunching destroyed!

Fortunately this does not happen because the electron and the wave *do not* travel with the same velocity. The (*u* − *c*) difference creates precisely the conditions for the microbunching to continue. In fact (Fig. 3 ▶, bottom), as the wave travels over a distance *L*/2 in a time *L*/(2*c*), the electron travels over a smaller distance *Lu*/(2*c*). The space shift between wave and electron is
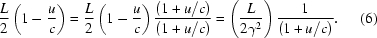
Using (2) and since *u* ≃ *c* and (1 + *u*/*c*) ≃ 2, we see that this shift is ∼λ/2, one-half wavelength! Thus, after one-half undulator period both the electron transverse velocity and the wave *B*-field are reversed, the Lorentz force keeps the same direction and microbunching continues.

This argument could be formulated in terms of phases: the difference between the electron oscillation phase and the wave phase stays constant. This is why we could so far neglect such phases (corresponding to the sine and cosine functions in the transverse velocity and in the wave), and analyze the phenomena with simple proportionalities.

## 7. Saturation

The above description, however, is not entirely realistic (Bonifacio *et al.*, 1984 ▶; Huang & Kim, 2007 ▶). As an electron gives energy to the wave, its own energy is lowered and its longitudinal speed decreases from *u* to (*u* − Δ*u*). Assume that the initial position of the electron with respect to the wave is favorable for the transfer of energy, *i.e.* that the directions of the electron transverse velocity and of the wave *E*-field produce negative work. The longitudinal speed decrease to (*u* − Δ*u*) changes these conditions and makes them increasingly less favorable for the energy transfer electron → wave.

As Δ*u* becomes bigger, at a certain point the electrons no longer give energy to the wave: instead, the wave gives energy to the electrons. This, in turn, increases *u* until the conditions for energy transfer from the electron to the wave are restored. Such a mechanism is repeated over and over: the energy oscillates between the wave and the electrons rather than continuing to increase exponentially for the wave (Dattoli & Ranieri, 1984 ▶). This is a key phenomenon underlying the saturation of the wave intensity amplification.

In order to estimate the conditions for saturation and in particular the ‘saturation length’ *L*_S_ (Bonifacio *et al.*, 1984 ▶; Huang & Kim, 2007 ▶) over which it occurs, we can start again from the energy transfer rate for one electron, proportional to *E*_W_ *v*_T_. So far we only considered amplitudes: but *E*_W_ (see Fig. 2 ▶) and *v*_T_ really are oscillating functions with their phases. We have already seen that
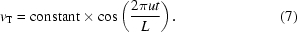
As far as the wave is concerned, we can write
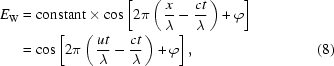
where ϕ is a constant phase angle. A linear change in speed from *u* to (*u* − Δ*u*) would modify the electron position at the time *t* from *ut* to approximately (*ut* − Δ*ut*/2), where the wave is proportional to cos[2π(*ut*/λ − Δ*u* *t*/2λ − *ct*/λ) + ϕ]. The difference between the two cosine arguments corresponding to *u* and to (*u* − Δ*u*) is πΔ*ut*/λ. When this difference becomes too big, the energy transfer conditions are reversed and saturation begins; this occurs for a difference value πΔ*ut*/λ related to 2π, *i.e.* for Δ*ut* ≃ 2λ.

Since Δ*u* << *u*, for *x* = *L*_S_ (the saturation length) *t* ≃ *L*_S_/*u*, and the same condition can be written,
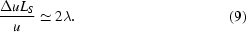
The speed decrease Δ*u* can be evaluated starting from the relativistic energy of the electron, γ*m*_0_ *c* ^2^ = *W*. By differentiating γ*m*_0_ *c* ^2^ = (1 − *u* ^2^/*c* ^2^)^1/2^ *m*_0_ *c* ^2^ with respect to *u*, this equation gives
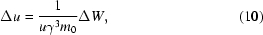
where Δ*W* is the energy loss, *i.e.* the energy given by the ‘average’ electron to the wave. Thus, (9) becomes
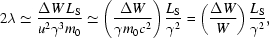
and therefore
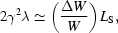
where (Δ*W*/*W*) is the fraction of its own energy that the ‘average’ electron gives to the wave. Using (2) we finally obtain
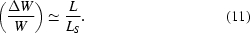
Generalized to all electrons, (11) implies that the ratio *L*/*L*_S_ approximately corresponds to the portion of the electron beam energy that is given to the wave before saturation occurs.

A closer look at the energy oscillation between the electrons and the wave enables us to make good use of (11) by calculating (Δ*W*/*W*). Consider once more the energy transfer rate, proportional to the product *E*_W_ *v*_T_. Taking for the wave and the transverse velocity the oscillating functions of (7) and (8), this product is proportional to
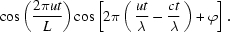
Using the elementary trigonometric property 2cos(α)cos(β) = cos(α + β) + cos(α − β), this expression is proportional to
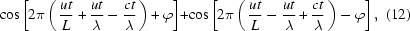
actually corresponding not to one oscillation only but to the superposition of *two* different oscillations. The argument of the second oscillation can be written as
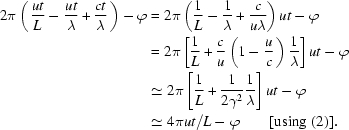
This is a rather fast oscillation whose effects average to zero and can be neglected in our discussion. With a similar procedure, the argument of the first term in (12) can be written as
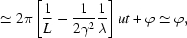
that, actually, does not correspond to an oscillation but to a constant.

However, we recover the oscillation by taking into account the speed change from *u* to (*u* − Δ*u*), so that the same term becomes
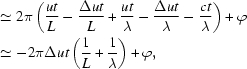
which, since *L* >> λ, is ≃ −2πΔ*ut*/λ + ϕ. This corresponds to an energy transfer oscillation with frequency 2πΔ*u*/λ, increasing as Δ*u* increases.

In essence, saturation does not occur initially because this energy oscillation frequency is low and only gain takes place, with the characteristic gain length *L*_G_. As the frequency increases, the gain length *L*_G_ becomes comparable with the electron path during one energy oscillation: there is no longer a steady gain and saturation is reached. This saturation criterion is equivalent to say (Murphy & Pellegrini, 1985 ▶) that the oscillation frequency becomes comparable with the gain rate given by (1), *u*/*L*_G_. We can therefore write
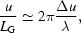
and, using for Δ*u* the result of (10),
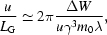
which gives
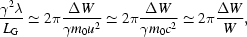
or, using (2),
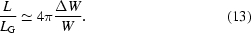
In terms of the FEL parameter ρ = 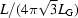, equation (13) implies that
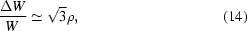
revealing another fundamental meaning of this parameter: it is a measure of the effectiveness of the overall energy transfer from the electrons to the wave. The conceptual physics background of rigorous theories (Bonifacio *et al.*, 1984 ▶; Huang & Kim, 2007 ▶; Murphy & Pellegrini, 1985 ▶) is consistent with (13) and (14) although the results have slightly different proportionality constants,
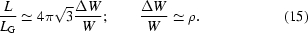
Equation (15) can also be interpreted with a somewhat different and interesting point of view: the stochastic wave emission changes the energy of each electron with respect to the others. This increases the energy spread until saturation occurs. The spread is related to the average energy loss Δ*W*, therefore (15) implies that ρ is also a measure (Murphy & Pellegrini, 1985 ▶) of the relative energy spread of the electron beam at saturation.

Combining (13) and (11), we finally obtain
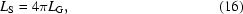
another interesting property of X-FELs, revealing the relation between the saturation length and the gain. Using (15) instead of (13), we obtain a version (Bonifacio *et al.*, 1984 ▶) of (16) with a more accurate proportionality constant,
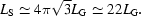

         

## 8. The underlying physics

The above discussion brings to light some of the fundamental physics facts in the X-FEL mechanism. In particular, it explains why it is more difficult to build free-electron lasers for X-rays than for larger wavelengths. Basically, for small wavelengths we need high-energy electrons, but high electron energy also increases the gain length, as shown by equation (4).

This brings us back to the apparent paradox that creating microbunches should be easier when they are spaced by a small wavelength, whereas in reality it is not. The paradox is solved by realising that this factor is more than offset by two others that clearly emerge from the above treatment. First, a large γ-factor negatively affects the transverse velocity, which is proportional to (*B*_0_ *L*/γ). Second, it impacts even more the longitudinal relativistic mass, proportional to γ^3^. In essence, the large γ-factor required for short wavelengths makes the electrons transversally and longitudinally ‘heavy’ and therefore difficult to move, negatively affecting both the individual-electron emission rate and microbunching.

As far as saturation is concerned, it is clear that the wave intensity amplification could not continue forever since at a certain point the electrons would run out of energy. This, however, is not an important feature: much before the electrons lose a substantial portion of their energy they slow down by emitting electromagnetic energy, change their phase with respect to the wave and start taking energy rather than giving it. Afterwards, the energy oscillates between electrons and wave rather than continuing to accumulate in the wave. Other effects also contribute to the saturation of the amplification (Milton *et al.*, 2001 ▶) making a full description more complicated.

## 9. Limitations

Table 1 ▶ summarizes the X-FEL properties that could be treated, at least semi-qualitatively, with our simple description. We note, however, that this approach is certainly not suitable for designing a real X-FEL and should not be applied beyond its limitations. First of all, we explicitly treated a planar undulator and did not consider helical insertion devices that are more effective for free-electron lasers (Bonifacio *et al.*, 1984 ▶; Huang & Kim, 2007 ▶). Furthermore, our analysis was performed in one dimension, without taking into account three-dimensional effects. Finally, an X-FEL requires very high amplification that is affected by several additional factors besides those we discussed. The corresponding treatment must be based (Milton *et al.*, 2001 ▶) on numerical solutions obtained with very sophisticated methods.

We can mention here the following additional factors affecting the amplification: electron energy spread, angular divergence, transverse electron beam size and diffraction of the wave. To a certain approximation their effects can be accounted for (Milton *et al.*, 2001 ▶) by multiplying the gain length by a ‘degradation factor’ χ > 1, so that the role of the parameters as described for example by equation (4) is still (at least qualitatively) valid.

The electron energy spread affects not only the amplification but also the saturation. In fact, amplification mainly starts with the optimal electron energy, whose γ-factor determines the wavelength [equations (2) and (3)]. But as the electrons transfer energy to the wave, their own energy decreases. The wave emission is not the same from all electrons, so that different electrons have different energies, with an increasing energy spread. At a certain point the energy spread is so large that there is no gain anymore. This saturation factor is combined and correlated to the previously discussed mechanism.

Other important issues were not treated at all here. We should mention at least the emission coherence and time structure. The coherence of the X-rays produced by a SASE X-FEL is very high laterally but limited longitudinally (Bonifacio *et al.*, 1984 ▶; Huang & Kim, 2007 ▶) because of the stochastic emission of the initial waves; this problem can be solved by seeding.

The time structure of the emitted beam is very interesting since it can reach the femtosecond and sub-femtosecond scale. Indeed, we have seen that the time duration of the emission by a single electron is *N*_u_λ/*c*. Taking typical values *N*_u_ ≃ 10^3^ and λ ≃ 1 Å = 10^−10^ m, this gives ∼0.3 × 10^−15^ s or 0.3 fs. The actual pulse length for a real X-FEL is influenced by several factors (Huang & Kim, 2007 ▶) that can also be used to control it. But the above basic time scale gives an idea of why the sub-femtosecond scale can be reached.

## Figures and Tables

**Table 1:** Summary of the properties of the different X-FEL parameters

Parameter	Symbol	Properties
Wave intensity	*I*	*I* = 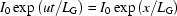
Emitted wavelength	λ	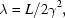 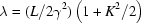
Undulator parameter	*K*	*K* = constant × *B*_0_*L*
Undulator bandwidth	Δλ	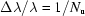
Electron transverse velocity	*v*_T_	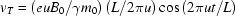 [proportional to 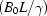 ]
Gain length	*L*_G_	*L*_G_ = constant × 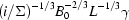
FEL (Pierce) parameter	*ρ*	ρ = 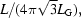 *ρ* = constant × 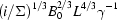
Energy transfer electron → wave	Δ*W*	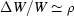
Saturation length	*L*_S_	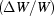 ≃ 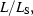 *L*_S_ = 4π 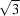 *L*_G_
